# Structure-Guided Approach to Identify Potential Inhibitors of Large Envelope Protein to Prevent Hepatitis B Virus Infection

**DOI:** 10.1155/2019/1297484

**Published:** 2019-09-04

**Authors:** Mahboubeh Mehmankhah, Ruchika Bhat, Mohammad Sabery Anvar, Shahnawaz Ali, Aftab Alam, Anam Farooqui, Fatima Amir, Ayesha Anwer, Saniya Khan, Iqbal Azmi, Rafat Ali, Romana Ishrat, Md. Imtaiyaz Hassan, Zarrin Minuchehr, Syed Naqui Kazim

**Affiliations:** ^1^Center for Interdisciplinary Research in Basic Sciences, Jamia Millia Islamia, New Delhi 110025, India; ^2^Department of Chemistry & School of Biological Sciences, Indian Institute of Technology Delhi, New Delhi 110016, India; ^3^Systems Biotechnology Department, National Institute of Genetic Engineering and Biotechnology, Tehran, Iran; ^4^Multidisciplinary Center for Advanced Research and Studies, Jamia Millia Islamia, New Delhi 110025, India

## Abstract

Hepatitis B virus (HBV) infection is one of the major causes of liver diseases, which can lead to hepatocellular carcinoma. The role of HBV envelope proteins is crucial in viral morphogenesis, infection, and propagation. Thus, blocking the pleiotropic functions of these proteins especially the PreS1 and PreS2 domains of the large surface protein (LHBs) is a promising strategy for designing efficient antivirals against HBV infection. Unfortunately, the structure of the LHBs protein has not been elucidated yet, and it seems that any structure-based drug discovery is critically dependent on this. To find effective inhibitors of LHBs, we have modeled and validated its three-dimensional structure and subsequently performed a virtual high-throughput screening against the ZINC database using RASPD and ParDOCK tools. We have identified four compounds, ZINC11882026, ZINC19741044, ZINC00653293, and ZINC15000762, showing appreciable binding affinity with the LHBs protein. The drug likeness was further validated using ADME screening and toxicity analysis. Interestingly, three of the four compounds showed the formation of hydrogen bonds with amino acid residues lying in the capsid binding region of the PreS1 domain of LHBs, suggesting the possibility of inhibiting the viral assembly and maturation process. The identification of potential lead molecules will help to discover more potent inhibitors with significant antiviral activities.

## 1. Introduction

Historically, one of the major human challenges has been to fight against infectious diseases; viruses are one of the causative agents of these diseases. Hepatitis B virus (HBV) infects 480-520 million people globally; this means that 1 out of every 12 people is affected, chronically [1]. Hepatitis B virus, in fact, causes about 650,000 deaths annually around the world. Sometimes, hepatitis leads to chronic liver disease and its recurrence may lead to liver fibrosis. Thus, it is the most usual cause of liver-related incidences and fatalities worldwide [2]. HBV is a small enveloped hepatotropic virus (~42 nm), having a partially double-stranded DNA genome (~3.2 kb) where the negative strand includes 3020-3320 nucleotides and the positive strand has 1700-2800 nucleotides [3, 4].

The HBV genome consists of four open reading frames (ORFs) encoding viral surface proteins (LHBs, MHBs, and SHBs also referred to as L, M, and S, respectively), polymerase (P), and core (C) and HBx (X) proteins. The LHBs has 389 amino acids (39 kDa) encoded by the PreS1, PreS2, and S domains. The MHBs antigen includes a polypeptide encoded by PreS2 and S, whereas the SHBs contains the polypeptide encoded by the S domain only [5, 6]. Depending on genotype, the PreS1 domain has 108, 118, or 119 amino acids, the PreS2 domain has 55 amino acids, and the S domain contains 226 amino acids, also known as hepatitis B virus surface antigen (HBsAg) [7]. Thus, LHBs is divided into three main domains: PreS1 (1-108), PreS2 (109-163), and S (164-389) [7]. LHBs also contains four putative transmembrane (I-IV) regions. According to previous reports, domains PreS1 and PreS2 have a very critical role in the viral entry process [8]. It has been proven that the PreS1 domain is required for HBV morphogenesis. Deletion of some amino acids between aa 114 and 163 of the PreS2 domain did not impair the production process of the virus [9]. Amino acids 2-78 of the PreS1 domain of the LHBs protein is involved in the recognition of a hepatocyte receptor [10]. The first 77 residues of the PreS1 domain are essential for HBV infectivity [11]. This protein also has the highly conserved “a” determinant region between aa 122 and 147 which remains involved in the binding of antibodies against HBsAg. A variety of mutations have been reported within this region [12]. Therefore, this region does not seem appropriate for drug designing.

Currently available targets for the anti-HBV therapeutic approach is largely based on nucleos(t)ide analogues (NAs) targeting polymerase. However, extensive usage of NAs is gradually becoming less effective due to many reasons, the most important is the emergence of resistant mutants and their consequences [13–18]. Finding HBV entry inhibitors and appropriate neutralizing antibodies are becoming the prime focus of therapeutic intervention [19]. Hence, the importance of identifying the structure of HBV proteins is being felt more than ever. Moreover, investigating the underlying immune mechanisms and associated signaling pathways, for instance Toll-like receptors, is also an important and growing concern these days. It is needless to say that targeting any of the envelope proteins could lead to a potential threat of a disproportionate accumulation of L, M, or S proteins. The accumulation of the L protein (LHBs) coordinates with tampering of the viral assembly process [20]. Its eventuality coincides with the generation of oxidative stress within the ER, consequently altering the downstream signaling processes. Under oxidative stress, the misfolding of proteins is a predominant phenomenon which is readily sensed by mammalian cells through a signaling network referred to as unfolded protein response (UPR). UPR is induced by factors known to contribute to calcium homeostasis and protein glycosylation and those related to physiological stresses like hypoxia and glucose deprivation [21]. Altogether, they disrupt the folding of proteins in ER, consequently triggering the signaling network which involves transmembrane protein kinases, transmembrane transcription factors, and transmembrane proteases. The cumulative response culminates into UPR activation [22]. The endoplasmic reticulum (ER) serves multiple functions needed for the execution of normal cellular function and cell survival. To name a few are Ca^2+^ storage, posttranslational modification, and the folding and assembly of newly synthesized secretory proteins. In the hepatitis B virus life cycle, the ER is the venue of envelopment as well as the maturation of viral particles [23, 24]. This cellular response triggers precursor mechanisms responsible for both survival and apoptosis [25]. Hence, both the advantages and disadvantages associated with potent inhibitors of the viral envelope protein, specially the LHBs, are naturally suspected.

Unfortunately, the 3D structure of the LHBs has not yet been discovered, and the critical functions of this protein are not fully determined. Thus, determining the structure of the LHBs protein can be very significant for researchers to clarify its function. In this study, we have tried to predict the structure of the LHBs of HBV by using bioinformatics instrument (computational methods) and accessible databases. The definition of the 3D structure of the large surface protein can be effective in controlling and preventing the development of hepatitis disease and hepatocellular carcinoma (HCC) and in exploring the better and comprehensive biological mechanisms and related signaling pathways involved in the HBV life cycle in liver cells. Sufficient knowledge of this protein structure may provide beneficial targets for designing some specific drugs for a better treatment of HBV infection. At the end of the present study, we introduce four optimized small molecules with less energy bonding and low toxicity. This study may thus lead to the identification of reliable candidate drugs for inhibiting HBV infection.

## 2. Materials and Methods

We have utilized modern computational methods to identify potential inhibitors of LHBs. The scheme of work is illustrated in the form of a flow chart (Supplementary, [Supplementary-material supplementary-material-1]).

### 2.1. Target Structure Prediction

The amino acid sequence of the LHBs of HBV (genotype D, subtype ayw) was retrieved from UniProt (P03138). The retrieved sequence was used to predict the secondary structure of LHBs by the PSIPRED tool [26] which predicts the ratio of *α*-helices and *β*-sheets within a protein from its sequence. This information is useful to generate a better structure in a 3D model. The ProtParam [27, 28] was used to identify the physicochemical characteristics such as the aliphatic index and GRAVY value to determine the hydrophobic or hydrophilic nature of the protein, which helps in estimating the chemical nature of the binding pockets of the protein. Furthermore, the tertiary structure of the protein was modeled by hybrid methods involving homology, threading, and *ab initio* approaches via structure prediction tools such as the RM2TS+ server [29] and I-TASSER [30]. RM2TS+ is one among the state-of-the-art prediction tools and derives its skeletal framework from the higher order Ramachandran map. I-TASSER utilizes the knowledge of structural templates from the PDB and generates models using iterative template-based fragment assembly simulations. These servers cover the exhaustive modeling algorithm in order to yield a promising tertiary model for the LHBs protein.

The structures obtained from RM2TS+ and I-TASSER were further analyzed for their quality check using the protein structure analysis and validation (ProtSAV) [31] and RAMPAGE tools [32]. The ProtSAV server can assess the quality of a predicted protein and can determine the correctness of the predicted model by giving the score. RAMPAGE generates the Ramachandran plot which defines whether the phi-psi value of each residue is in the allowed or disallowed location [33]. Both tools helped in analyzing if the tertiary predicted model of the LHBs protein is within the acceptable limits of the structure. The best models obtained based on the ProtSAV score and percentage allowed residues using ProtSAV and RAMPAGE, respectively, were further optimized using Galaxy refine tool. After several refinements, the 3D structure was minimized using AMBER 14 [34]. The protein was provided with a water environment of 12 Å TIP3P water model. After minimization, slow heating for 20 ps was run and the system was left in the NPT ensemble for a 20 ns run length to study its most favorable structural conformations to yield a final model of the LHBs protein.

### 2.2. Binding Site Prediction

The identification of active sites and experimental information about LHBs is essentially important. In order to predict the active site of the refined model, the AADS server [35] was used. AADS is a tool to predict all the possible binding pockets within a protein based on its tertiary structure with a 100% accuracy of acquiring the real binding site within the top 10 identified pockets. The final best-modeled protein obtained was submitted to the AADS server (http://www.scfbio-iitd.res.in/) which then identified the best ten potential active sites.

### 2.3. Hit Identification

After the binding sites of the target protein are predicted, the RASPD software [36] was used to identify the best hits from a library of a million small molecules obtained from ZINC database [37]. The protein was screened against all of the best ten binding sites using RASPD. The hit molecules were further optimized based on the Lipinski parameters such as the number of the hydrogen bond of acceptors and donors, Wiener index, volume for the protein and functional groups, and the molar refractivity and through proper ADMET profiles. The most interesting feature of RASPD is that it generates a set of hit molecules based on the complementarities of the properties. The screening was done against the million-molecule database which resulted in more than 1000 hits per binding site, out of which the top 50 hits for each site was taken for further analysis.

### 2.4. Molecular Docking

The screening is followed by all atom energy-based Monte Carlo protein-hit molecule docking using ParDOCK [38] for identifying the best candidates which could be selected for empirical synthesis and testing. The ParDOCK module of Sanjeevini is an automated server for protein ligand docking (http://www.scfbio-iitd.res.in). It considers the optimal position of ligands with the best configuration in binding sites of the target protein and classifies them according to their interaction energies. Thus, the best molecule is chosen based on the score of the binding energy of candidates and it is considered as the best binder to the target. The threshold kept here was -11 kcal/Mol binding energy values for the L protein-small molecule complexes.

### 2.5. Molecular Dynamic Simulations

From static, the energetic perspectives in terms of dynamics were taken into consideration by running 100 ns long molecular dynamic simulations mimicking the *in vitro* environment to increase the reliability of these hits as potential inhibitors and to understand their mode of interaction mechanisms. The complexes were solvated in TIP3P [39] water box molecules of 12 Å. The input for simulations was the best-docked protein inhibitor generated via ParDOCK for each inhibitor. Ligand and protein files were prepared using the AMBER 14 package for performing MD. Parameter and topology files were generated using the “ff99SB” and “GAFF” force field, respectively [40, 41]. The compounds were first subjected to 5000 steps of minimization (2500SD+2500CG) to set the water box. A further 5000 steps of hydrogen minimization (2500SD+2500CG) were performed on the complex to relieve any steric clashes. Slow heating of the solvent to 300 K over a period of 20 ps was done. Equilibration for 300 ps was also performed before letting the manufacture phase run for 100 ns with a time step of 2 femtoseconds under NPT conditions with boundary situations. Simulations were investigated through energy and density plots. The amount of pressure and temperature was kept fixed, and hydrogen atoms were finite by the SHAKE algorithm [42, 43]. The poses were written after every 100 ps. Finally, analysis of the molecular dynamic curves was carried out and PME summation [44] was used for electrostatic calculations. All the results presented here were analyzed on the last 100 ns trajectory of each system.

### 2.6. Toxicity Prediction

Pharmacokinetic properties and percent human oral absorption values such as absorption, distribution, metabolism, excretion, and the potential toxicity (ADMET) of the selected molecules were estimated with the admetSAR database [45], SwissADME [46], and Komputer-Assisted Technology (TOPKAT) software [47] developed by Health Designs Inc. (Discovery Studio 2.5, USA). These values provide an estimate for the drug likeness of the candidate molecules and predict their bioavailability.

## 3. Results

### 3.1. Physicochemical Characteristics

To identify the physicochemical characteristics of LHBs, the ProtParam tool was used (Supplementary, [Supplementary-material supplementary-material-1]). The isoelectric point value (pI = 8.40) shows that the target protein is basic in nature. The half-life of LHBs is approximately 30 hours, and its stability lies in the middle (46.08). The aliphatic index determines the relative volume occupied by a protein and its aliphatic side chains, and the results show that LHBs has a high aliphatic index (82.24). The GRAVY value is the sum of the hydropathy values of all the residues of the protein divided by the number of amino acids of that protein, and based on this, the LHBs is considered a hydrophobic protein (0.146).

### 3.2. Protein Structure Prediction and Validation

**Figure 1:**
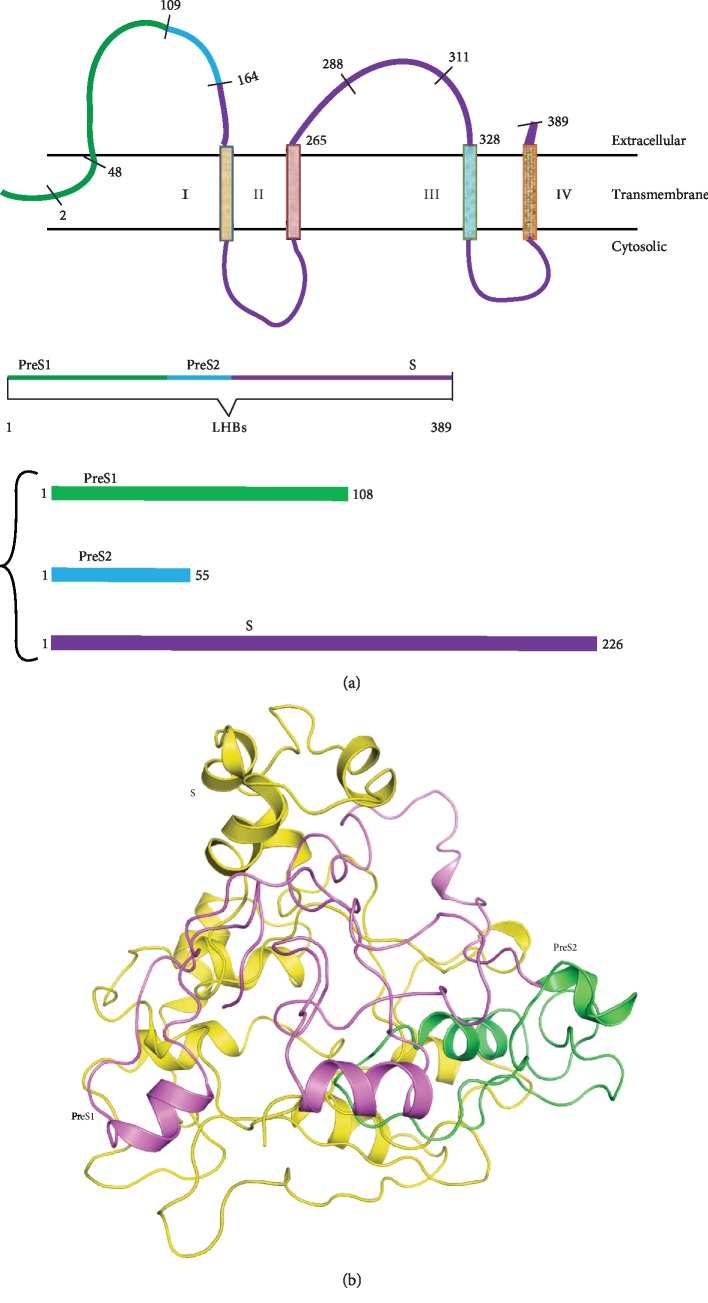
Schematic representation of the modelled protein structure of the large surface protein of HBV (LHBs). (a) Numbering and positions of amino acid residues as determinants of PreS1, PreS2, and S domains; extracellular, transmembrane, and cytosolic regions as determinants of the structure of LHBs. (b) The modeled structure of LHBs showing the PreS1, PreS2, and S domains in magenta, green, and yellow colors, respectively.

For predicting the secondary structure of LHBs, the amino acid sequences were submitted to the PSIPRED server. The large envelope protein is 389 residues long, of which 26% is *α*-helix, 9% is *β*-sheet, and approximately 60% is coil(Supplementary, [Supplementary-material supplementary-material-1]). The amino acid sequences were exported to RM2TS+ and I-TASSER software which uses both homology and *ab initio* approaches for generating three-dimensional structures (Supplementary, [Supplementary-material supplementary-material-1]). The predicted structure of LHBs was refined using “Galaxy refine,” and energy was minimized using AMBER 14; MD simulations were run for 100 ns. A single polypeptide of LHBs with its boundaries determining the domains PreS1, PreS2, and S and with its residues lying within extracellular, transmembrane, and cytosolic regions has schematically been represented in Figure 1(a). The modeled structure of LHBs is shown in Figure 1(b). The refined structure has a close resemblance to the predicted secondary structure. Figure 1(b) shows three distinct domains of LHBs, also referring to the corresponding surface proteins of HBV.

**Figure 2:**
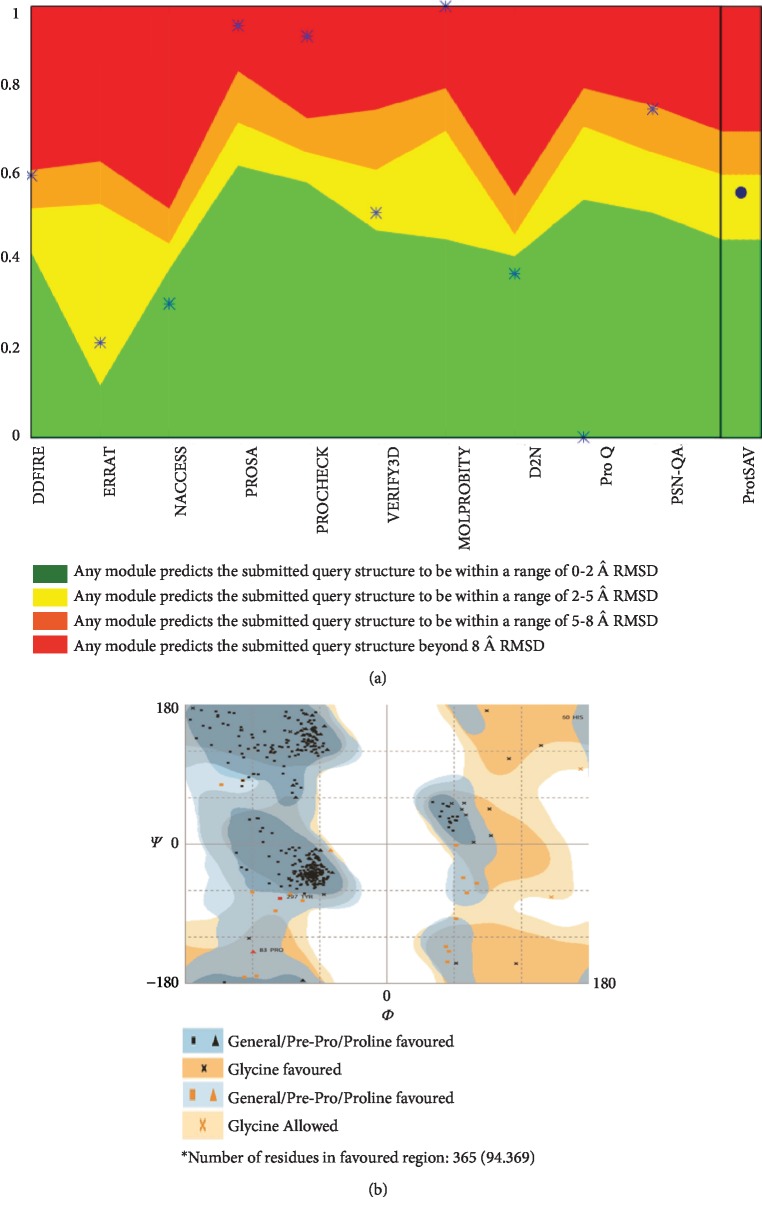
(a) ProtSAV analysis: the modelled LHBs protein lies in the yellow region. (b) RAMPAGE analysis: 94.39% of the residues of LHBs lie within the favored region.

After simulations, the structure was further analyzed by the structure validation tool “ProtSAV.” According to the ProtSAV protocol, green is the best and the yellow color is acceptable; our modeled protein appeared in the yellow region (Figure 2(a)). Furthermore, we calculated the Ramachandran plot (RAMPAGE tool) for structural quality; we found that 94.39% of the residues are in the favored region (Figure 2(b)).

To predict the best structure of a target protein and to design especially small molecules based on drug discovery, we required accurate information about the binding pocket on the target which can inhibit the protein function. The prominent binding sites of the LHBs protein were evaluated through AADS which identified 19 potential binding sites on the LHBs structure and out of which we considered the top 10 cavities.

### 3.3. Hit Identification and Molecular Docking

For finding the probable hits, the top 10 cavities of the structure which were identified by AADS were subjected to the RASPD software. The RASPD provides more than 1000 molecules against the predicted cavities of our target protein by selecting the ZINC database (-7.0 kcal/Mol to -13.5 kcal/Mol). The top 50 compounds of each cavity were taken for further scoring analysis and docking. Furthermore, all cavities were docked with compounds proposed by RASPD through the ParDOCK software. It imports the ligands with the best configuration in the target binding site and scores them based on their estimated free interaction energies using the BAPPL scoring function [48]. Finally, six compounds were selected based on the least binding energy between -11 kcal/Mol and -12.69 kcal/Mol. These were further subjected to MD simulation to check for the protein-hit interactions of over 100 ns.

### 3.4. MD Simulation

**Figure 3:**
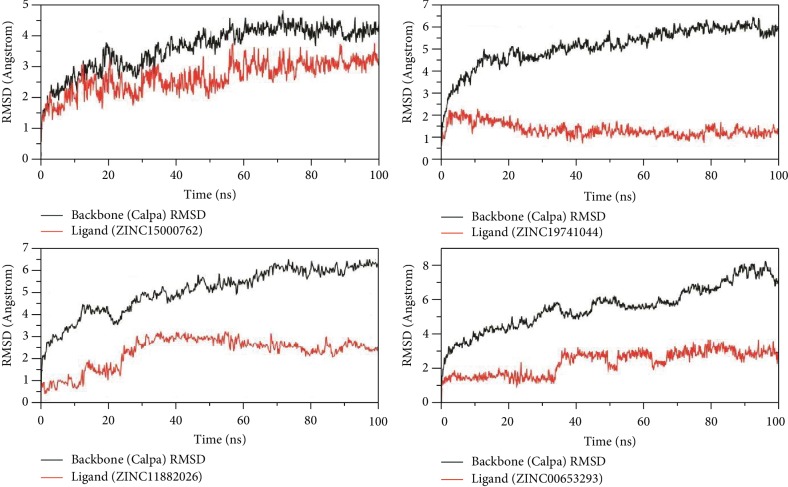
RMSD plots of the LHBs protein with corresponding ligands.

All molecular dynamic simulations were carried out using AMBER 14. The overall binding free energy of the protein-hit molecule complexes throughout the 100 ns trajectories was calculated using BAPPL. To calculate the overall binding free energy between the targeted LBHs protein and the hit molecules over 100 ns long explicit simulations, the binding energies of the frames that were obtained at an interval of each nanosecond were calculated and then averaged out. A threshold of -8.0 kcal/Mol overall binding free energy for 100 ns simulations was set for proposing the molecules as potential therapeutics against the LHBs protein. Finally, we found four out of the six screened hit molecules as potential inhibitors based on the binding free energy score during molecular dynamic simulations (Table 1). For each system simulated, the root mean squared deviation, energy (kinetic, potential, and total), density, and temperature were monitored to ensure that the standard deviation of each of these values was within acceptable limits of experimental error. The RMSD graphs of the hit molecules with promising inhibitory potential along with the above values are shown in Figure 3, which shows an overall stability within the complex. Finally, we found four out of the six compounds as potential inhibitors based on the affinity score and explicit simulation (Table 1, [Supplementary-material supplementary-material-1]).

**Figure 4:**
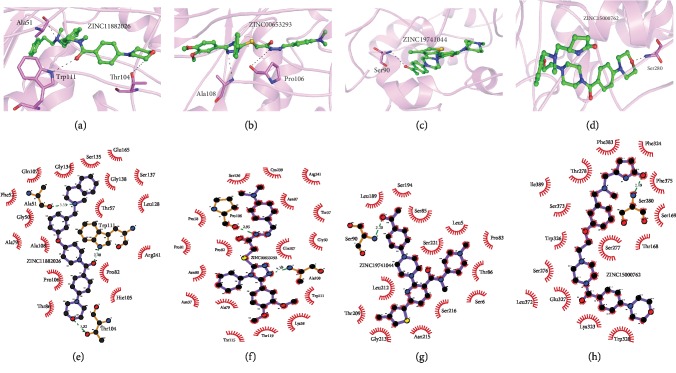
The binding patterns (a–d) and 2D representations (e–h) of LHBs in-complexed with ligands are shown: (a, e) 11882026, (b, f) 00653293, (c, g) 19741044, and (d, h) 15000762.

We visualized the interactions of our LHBs vs. potential ligands (complexes) using PyMOL and LigPlot (2D) (Figure 4). Our results indicate that all four selective ligands are bonded to the amino acids which are among the important regions, primarily at nucleocapsid binding residues and at a residue relevant to the entering of the virus and consequent infectivity. ZINC11882026 has been bonded by three H-bonds through Ala51 (3.33 Å); Thr104 (3.32 Å) of LHBs, which is in PreS1 (1-108); and Trp111 (3.08 Å) of LHBs; which is in PreS2 (109-163). Both of these regions are more important for NTCP receptor-mediated entry and nucleocapsid binding, respectively [8, 47, 48]. The compound ZINC00653293 is involved with Pro106 (2.9 Å) and Ala108 (3.2 Å) of LHBs; both of them are in PreS1 that signifies the nucleocapsid binding and consequent envelopment of a virus in the ER. The compound ZINC19741044 has only one H-bond with Ser90 (3.2 Å) of LHBs, which is also in the PreS1 region. The compound ZINC15000762 binds with one H-bond to Ser280 of LHBs (2.89 Å) linked to the protein which lies on the external side of the viral envelope ([Supplementary-material supplementary-material-1]). Interestingly, none of them connects to the amino acids in the “a” determinant region (aa 122-147 of SHBs, corresponding to aa 286-311 of LHBs, respectively). Therefore, in case of any mutation in this region, selective ligands will still be suitable for LHBs inhibition. Therefore, our results indicate that all four selective ligands are bonded to the amino acids which are among the important regions referring to the process of entry and viral maturation. For more details, the molecular properties of each compound were also extracted from the ZINC database (http://ZINC.docking.org/), and the results indicate that our selective compounds have pharmaceutical capabilities (Table 2).

### 3.5. Drug Likeness Prediction

Drug ability and toxicity of selected candidates have been computed by using admetSAR, SwissADME, and TOPKAT software. Parameters such as absorption, distribution, metabolism, excretion, and the toxicity [49–51] of selected candidates were evaluated by admetSAR ([Supplementary-material supplementary-material-1]). All candidates showed positive results for the blood-brain barrier, Caco-2 permeability, and human intestinal absorption, warranting that they have no side effects about absorption. Also in terms of metabolism, various substrates and inhibitors of cytochrome P450 [52, 53] were investigated and the results are indicated in [Supplementary-material supplementary-material-1]. In case of toxicity, all four compounds have non-AMES toxicity, are noncarcinogenic, and have no carcinogenicity (three-class) required. To distinguish any unfavorable toxic properties of novel hits, TOPKAT software was also used [54]. Several descriptors such as the Ames mutagenicity test, weight of evidence carcinogenicity (v5.1) (WOE), rat oral LD50, skin irritation, skin sensitization, and aerobic biodegradability [55, 56] were investigated ([Supplementary-material supplementary-material-1]).

The TOPKAT results indicated that none of the compounds were mutagenic, and all of them showed a negative response to the Ames mutagenicity test. The weight of evidence carcinogenicity (WOE) was also evaluated as another toxicity predictor to determine the carcinogenicity of virtual hits, and all compounds were found to be noncarcinogenic, except compound (ZINC19741044). About skin irritation and skin sensitization, all four candidates have no skin irritancy and no skin sensitivity effect, and of these compounds, only compound ZINC00653293 and ZINC15000762 were predicted to be aerobic biodegradable. The comparative ADMET data and TOPKAT results of virtual hits with a standard drug proposed that selected hits may be used as inhibitor molecules for HBV.

## 4. Discussion

### 4.1. Structurally Validated Modeled LHBs Occupy Significant Numbers of Residues in the Favored Region

The surface open reading frame (S-ORF) of the hepatitis B virus genome encodes proteins of three distinct types; namely, large (L), middle (M), and small (S) surface proteins, also referred to as LHBs, MHBs, and SHBs, respectively. All of them share common C terminals [5]. The S protein is also referred to as HBsAg and is the most abundant among these three proteins. Its expression is not directly dependent on the L and M envelope protein expression. However, in order to accomplish the normal viral life cycle, the presence of the proper proportion of all the three envelope proteins is essential. If LHBs is overexpressed, an indirect hindrance appears in HBsAg secretion [57]. In spite of many studies performed on the pathogenicity of the large HBV surface protein (LHBs) over the past two decades, there is still not much information about the 3D structure of this protein. Several evidences indicate that LHBs is expressed in liver cells and plays a prominent role in chronic hepatitis B even in the development of HCC. On the other hand, with regard to the realization of this fact that the fundamental role of this protein in vaccine development is undeniable, our motivation to predict the 3D structure and perform a geometry optimization was conceived more than ever.

In this study, the LHBs of HBV genotype D has carefully been modeled with the aid of RM2TS+ and I-TASSER and subsequently has the best possible refinements. The purpose of the selection of genotype D is that it is the most prevalent genotype among the Indian population. The amino acid sequences of LHBs were elaborately analyzed, and efforts were made to predict the best optimized structure. The potential LHBs structure was subjected to ProtSAV and RAMPAGE. Thus, the obtained structure of LHBs lies in the acceptable yellow region (Figure 2(a)) with 94.39% of the residues occupying the favored region (Figure 2(b)). Our findings provided basic insights into the 3D structure of LHBs and introduced four novel compounds having the best interconnection with the most important structural and functional regions of this protein. It is important to mention that LHBs performs many different functions, right from the viral entry to the hepatocytes until the maturation of infectious virions within the endoplasmic reticulum (ER). The protein facilitates the adherence of infectious virions to the heparansulphate proteoglycan on the surface of hepatocytes. The myristoylated N-terminal of the PreS1 domain of the L protein subsequently binds to the sodium taurocholate cotransporting polypeptide (NTCP), the potent receptor identified during the last few years [58]. The PreS1 sequence (2-48) specifically interacts with NTCP [8, 59]. Furthermore, aa 49-75 is also needed for infection. The definitive function of this sequence is not fully established, but it has widely been assumed that aa 49-75 is involved in targeting the NTCP [59]. There is a spacer region towards the C-terminal to the NTCP binding site which consists of a nucleocapsid binding sequence, essentially needed for the envelopment process of HBV [8].

### 4.2. Identification of Potential Compounds That Possess the Best Features of Binding Affinity with LHBs

The present study has elaborately been undertaken to create a reliable model to display the construction of LHBs using exhaustive computational approaches, and subsequently, identification of potential compounds showing high affinity with the LHBs protein was carried out. The interactions of LHBs vs. potential ligands (complexes) were visualized in PyMOL (3D) and LigPlot (2D). Four selective ligands, ZINC11882026, ZINC19741044, ZINC00653293, and ZINC15000762 were identified establishing covalent linkages with the amino acids significantly essential for the viral entry process and viral nucleocapsid envelopment. To the best of our knowledge, these compounds as well respective amino acids have for the first time been explored in this study, which apparently could be of relevance in antiviral drug design and related studies. The obtained insight into the modeled protein and the identification of potential inhibitory compounds could be an important milestone in search for the alternative therapies against HBV infection.

The structural organization of L, M, and S proteins and their consequent functions, on one hand are guided in an orchestrated manner, and on the other hand, these critical sequences and structural organization provide enormous space to explore the newer therapeutic approaches for the improvement in HBV-related disease management over a period of time. Interestingly, all the four ligands show a desired affinity with the NTCP binding region (aa 2-48), the most important newly discovered entry receptor. The one which has the closest proximity and forms a hydrogen bond at Ala51 (3 aa downstream) is ZINC11882026. It is perceived that blocking Ala51 with the help of this compound may have some influence on inhibiting the viral entry process. The rest of the residues (111, 104, 106, 108, and 90) are being targeted by ZINC11882026, 00653293, and 19741044 (Figure 4 and [Supplementary-material supplementary-material-1]). It is quite interesting to point out that these residues are lying within the so-called spacer region of LHBs, which is supposed to be involved in NC binding that is the envelopment and maturation process of the encapsidated viral genome undergoing in the ER. In the present study, we were able to identify the ligands targeting the spacer region of LHBs for the first time. In other words, the envelopment and morphogenesis of the virus could potentially be inhibited with the help of these ligands.

Only the receptor inhibitor Myrcludex B at a median contraction of 80 pmol/L has been known till date. It is a synthetic N-acylated PreS1 lipopeptide and has been shown to block the NTCP and virus entry process, both *in vitro* and *in vivo* [60, 61]. There was lack of information regarding capsid binding inhibitors. The present study exhaustively searched, analyzed, and identified three inhibitors at the capsid binding locus of LHBs and one at the suspected extension of the receptor. The combination of these compounds in varying permutation combinations could be a novel approach for the simultaneous inhibition of entry/infection and capsid binding/envelopment, thus, targeting the early as well as later phases of viral infection at the same time. However, these warrant an urgent need of testing these newly identified compounds singly or in combination in a cell culture system.

### 4.3. The Binding Affinity of Identified Compounds and Regulation of Cellular Redox Homeostasis-Associated Therapeutic Advantages Might Outnumber the Disadvantages

Careful modeling of LHBs followed by the identification of entry as well as capsid binding inhibitors having maximum suitability to be used as potential drugs might be of important significance in search of newer therapeutic approaches in order to overcome the difficulties associated with extensive use of nucleoside analogues. However, the suspected accumulation of disproportionate levels of the large surface proteins, in the absence of capsid binding and assembly of the virus, and thus, the possible downstream consequences like the generation of oxidative stress and activation of resultant pathways, at this stage cannot be ruled out. Three of the four inhibitors, namely, ZINC11882026, ZINC00653293, and ZINC19741044, make hydrogen bonds with amino acid residues lying in the capsid binding region of the PreS1 domain of LHBs (Figure 4 and [Supplementary-material supplementary-material-1]). The expected result of the usage of these inhibitors may coincide with the viral assembly process. The logical consequences could lead to the accumulation of LHBs within the ER and the possible eventuality could also be related to the disproportionate existence of the L, M, and S envelope proteins intracellularly. The cumulative effect might be reflected in the generation of oxidative stress. It is of quite relevance to critically apprehend the advantages as well as disadvantages of the potential inhibitors. Nevertheless, experimental validation would essentially be needed.

As reported earlier, viral infection may trigger the UPR as a consequence of the overloading of the ER. Viral infection-mediated ER stress, on one hand may promote cell suicide as a strategy to avoid viral replication and spreading, while on the other hand may contribute to innumerable deleterious consequences [62, 63], primarily related to the production of overwhelming levels of reactive oxygen species (ROS). The predominant forms are anionic superoxide (O_2_^−^) and hydrogen peroxide (H_2_O_2_) [64, 65]. We apprehend a couple of possible consequences in response to the binding affinity shown by potential compounds with the LHBs identified in this study. Firstly, the possibility of a decreased or impaired encapsidation process might enhance the accumulation of LHBs and/or MHBs in the ER. As a result, the generated stress could be a major contributory cause to the production of more H_2_O_2_. This could lead to an increased number of disulfide bridge formation during the folding of a protein, involving enzymes such as endoplasmic reticulum oxidoreductin-1 alpha (Ero1*α*). The relevant signaling pathway might get triggered consequently. Secondly, in response to stress signals, Ca^2+^ might be released from the ER and taken up by mitochondria via the mitochondrial uniporter (MCU). The point of contact between the ER and mitochondria has a major role to play while triggering Ero1*α*/Ca^2+^ in this signaling pathway. The activated pathways might involve mitochondrial accumulation of Ca^2+^ in order to trigger signaling and downstream processes [22]. The point of contact between the ER and mitochondria is established by the inositol triphosphate receptor (IP3R) and the voltage-dependent anion channel protein (VDAC). There is a direct impact on the functioning of the electron transport chain (ETC) by the Ca^2+^ level inside the mitochondria with consequences of an increase in ROS production [22].

We had previously demonstrated in one of our studies the intracellular reactive oxygen species production culminating into mitochondrial depolarization. However, the study was an elaborated comparative account of hepatitis B virus X protein mutants K130M, V131I, and KV130/131MI to investigate their roles in fibrosis, cirrhosis, and hepatocellular carcinoma. It was found that the expression of KV130/131MI induced cell proliferation and altered the expression of cell cycle regulatory genes in favor of cell proliferation, intracellular reactive oxygen species (ROS) production, and mitochondrial depolarization [66]. Hence, it is quite understandable that the present study highlighting the binding affinity of newly identified compounds with LHBs might enhance ROS production through Ca^2+^-mediated alterations in mitochondrial functioning and unfolded protein response.

However, the activation of the Nrf2/ARE pathway is a parallel cascade of signaling in order to provide the rescue operation sensed through the deleterious effects of ROS. The hepatitis B virus induces this pathway of antioxidant defense [20, 67]. Both the transfected cell lines with the HBV genome as well biopsy samples from CHB patients have demonstrated the salvage pathway. Several independent studies have shown the increased levels of Nrf2-dependent phase II enzymes such as glutathione synthetase (GSS) and glutathione reductase [24, 68]. The activation of Nrf2/ARE is known to be triggered by HBx and LHBs also in in vitro cell cultures [67]. PERK, one of the transmembrane kinases, is very important in PERK-dependent activation of Nrf2 and is critical for survival signaling. PERK-dependent phosphorylation leads to the nuclear accumulation of Nrf2 and increased transcription of Nrf2 target genes [69]. PERK function is needed for cellular response to ER stress [23]. Nrf2 confers a protective advantage to stressed cells with the help of an interesting rescue operation that its activation contributes to the maintenance of glutathione levels. Glutathione functions as a buffer/neutralization component at the time of accumulation of reactive oxygen species during the unfolded protein response. Logically, the deleterious effects of Nrf2 or PERK deficiencies could be attenuated by the restoration of cellular glutathione levels or Nrf2 activity.

Based on the findings indicating the appreciable affinity of the inhibitor compounds identified in this study and their high degree of suitability as a drug seen with the help of reliable computational methods (Tables [Supplementary-material supplementary-material-1] and [Supplementary-material supplementary-material-1]), these compounds could be of great importance in the future. However, it is difficult to predict if these compounds would act as an antiviral either by suppressing the viral load directly or by reducing the opportunity for newer infection of the uninfected hepatocytes in *in vitro* conditions (transformed cell lines). Again, whether the magnitude of ER stress and generation of ROS would be substantial or there will be no or only a minimal level of such stress due to the apprehended accumulation of LHBs cannot be predicted at present.

It must be emphasized here that all the previous studies and their findings discussed here are based on several types of point as well as deletion mutants within the S-ORF, more specifically in the functionally critical residues of the PreS1 and PreS2 domains of LHBs. Several mutations in S-ORFs lead to ER stress in hepatocytes through the accumulation of HBV virions and in the general consequent development of hepatocellular carcinoma and liver damage. Most studies have employed specific types of deletion mutants in LHBs [70, 71]. It is important to mention here that mutations disturbing amino acids 88-108 of PreS1 and the first five amino acids of PreS2, where most of the newly identified compounds in our present study are supposed to interact, have been believed to varyingly influence the capsid binding with the eventuality of interference with HBV assembly. However, the M protein-deficient HBV consisting of arbitrary PreS2 sequences within the L protein has been shown to be infectious [72]. However, because of the presumed lack of viral assembly due to a deficient M protein, the sustainability of the infection still remains unanswered.

Based on the available literature, we still believe that the identified compounds in the present study would be potential inhibitors, primarily for the residues lying within the nucleocapsid binding region at the carboxy terminal region of PreS1 of LHBs (aa90, 104, 106, 108, and 111). The most effective compounds, referred to as ZINC11882026, might have the best inhibitory effects both by virtue of establishing a covalent bond at aa 51 (a suspected residue at the locus within PreS1 facilitating the entry process) and by interacting with aa 104 and 111, which are responsible for NC binding and viral maturation. Both of the significant processes of the viral life cycle, one at the early events of infections and another at the late events related to viral maturation and morphogenesis, may simultaneously be blocked hand in hand with this compound. It is theoretically conceived that ZINC11882026 may regulate both the early and late phases of viral life cycles, thereby controlling the overaccumulation of LHBs and other viral proteins within the ER and cytosol. This could also be attributed to the regulation of the deleterious effects of ROS production and oxidative stress generation; consequently, signaling pathways responsible for tumor genesis might not cross the limits that generally accelerate the process of liver damage. Furthermore, the combination of compound ZINC11882026 with any of the other three compounds could also be of significant relevance in inhibiting the viral life cycle without deregulating the underlying molecular mechanisms pertaining to the signaling cascades of oncogenesis and/or apoptosis by maintaining the desired proportion of L, M, and S proteins. The fourth compound with ZINC15000762 has been shown to interact with serine aa 280 of LHBs which is in fact the aa 117 of the major surface protein (SHBs or HBsAg). This residue lies within the well-known major hydrophilic region (MHR) of HBsAg (aa 100-160). Dual possible impacts may be attributed by this inhibitor: the first is viral infectivity and the other could be its influence on the antigenicity of HBsAg. The region is also known as the antigenic loop (AGL), and researchers have demonstrated that the residues of AGL, transmembrane-II (Trn-II), and transmembrane-III (Trns-III) are required for infectivity, particularly Gly-119, Pro-120, Cys-121, Arg-122, and Cys-124 [62]. However, the identified residue Ser-117 (Ser-280 with reference to LHBs) in thus study, being present in AGL, might have a role in infectivity also. However, it may not have as much significance with respect to antigenicity like the other residues of MHR. It is quite logical to appreciate that, in addition to many other important features, a good antiviral must have the primary quality of being a good inhibitor of the viral life cycle without significantly modulating antigenicity, particularly for these viral proteins which are involved in eliciting the host immune response. In light of the concept, the inhibitor ZINC15000762 establishes covalent linkage with Ser-117 (with reference to SHBs or HBsAg), and although it would not be able to influence the antigenicity of HBsAg substantially, it may inhibit the infectivity process, in accordance with previous studies. The resulting situation would possibly be a favorable situation in order to present the infectious virions or subviral particles to the immune response of the host and consequently eliminate the existing infection with simultaneous inhibitions of newer infectivity. Nevertheless, the biochemical significance of the amino acid (ser-117) and its presence in AGL could be of relevance to prevent infectivity if inhibited by ZINC15000762.

To conclude, the LHBs modeled with utmost precision and care and the identification of biologically potent inhibitors with the best possible therapeutic features are novel findings, which possibly could be of positive impact in the search of newer therapeutic approaches against hepatitis B virus infection. The expected ROS generation and ER stress development could be managed by using the combination of compounds in order to maintain the proportionate expression and accumulation of large, major, and middle surface proteins, for the purpose of accomplishing normal encapsidation and envelopment processes as generally seen in wild-type HBV.

## Figures and Tables

**Table 1:** The binding affinity of top hits with LHBs are shown.

ZINC ID	ParDOCK ID	RASPD score (kcal/Mol)	ParDOCK score (kcal/Mol)	Average affinity^∗^	Proposed
ZINC11882026	33625775	-11.4	-11.49	-8.87	Yes
ZINC00653293	21425236	-13.1	-11.75	-8.60	Yes
ZINC19741044	72860345	-13.4	-12.69	-8.33	Yes
ZINC15000762	78876711	-10.1	-12.46	-8.71	Yes
ZINC11784805	34135187	-10.5	-12.36	-4.63	No
ZINC12243260	32993271	-11.5	-12.39	-2.88	No

^∗^Found for 100 ns scale explicit simulations.

**Table 2:** Molecular properties of selected ligands.

Compound ID	*A*Log*P* (≤5)	Molecular weight	H-bond acceptors (≤10)	H-bond donors (≤5)	Apolar desolvation (kcal/Mol)	Polar desolvation (kcal/Mol)	Rotatable bonds (≤10)
ZINC11882026	3.78	514.69	6	1	-1.52	-49.29	9
ZINC00653293	1.91	515.614	9	0	-5.09	-14.77	6
ZINC19741044	4.02	532.734	7	1	13.34	-52.43	6
ZINC15000762	1.70	536.697	9	2	7.87	-57.67	10

## Data Availability

The amino acid sequence of LHBs of HBV (genotype D, subtype ayw) was retrieved from the UniProt (P03138). The data for the ligands were obtained from the ZINC database. These data and other data used to carry out the study and to support the findings of this study are included within the article and in the supplementary information files.

## Supplementary Materials

Supplementary Materials Figure S1: diagrammatic representation of a structure-guided approach to design potential inhibitors of the LHBs protein. Figure S2: distribution of *α*-helix (pink), *β*-sheet (yellow), and coil (black) in the LHBs protein. Figure S3: the structures which were obtained from RM2TS+ and I-TASSER were further analyzed using various tools such as ProtSAV and Protein Structure Analysis and Validation, but in order to get a better structure, the MD was run for 20 ns. Table S1: physiological properties of the LHBs protein. Table S2: 2D structure of the identified ligands from the ZINC database. Table S3: comparative analysis of hydrogen bonds in LHBs and four ligands. Table S4: computationally predicted ADME properties of the identified compounds 1-5 by admetSAR and SwissADME. NS: nonsubstrate; S: substrate; NI: noninhibitor; NT: nontoxicity; NC: noncarcinogen; NR: not required. Table S5: TOPKAT values of the identified compounds. NM: nonmutagen; NC: noncarcinogen; C: carcinogen; NI: nonirritant; NS: nonsensitive; S: sensitive.
