# Supplementary material for: Structure-Guided Approach to Identify Potential Inhibitors of Large Envelope Protein to Prevent Hepatitis B Virus Infection
Source: Oxid Med Cell Longev. 2019 Sep 4;2019:1297484. doi: 10.1155/2019/1297484 (PMC6854180; doi:10.1155/2019/1297484)
Supplement: Supplementary Materials — Figure S1: diagrammatic representation of a structure-guided approach to design potential inhibitors of the LHBs protein. Figure S2: distribution of α-helix (pink), β-sheet (yellow), and coil (black) in the LHBs protein. Figure S3: the structures which were obtained from RM2TS+ and I-TASSER were further analyzed using various tools such as ProtSAV and Protein Structure Analysis and Validation, but in order to get a better structure, the MD was run for 20 ns. Table S1: physiological properties of the LHBs protein. Table S2: 2D structure of the identified ligands from the ZINC database. Table S3: comparative analysis of hydrogen bonds in LHBs and four ligands. Table S4: computationally predicted ADME properties of the identified compounds 1-5 by admetSAR and SwissADME. NS: nonsubstrate; S: substrate; NI: noninhibitor; NT: nontoxicity; NC: noncarcinogen; NR: not required. Table S5: TOPKAT values of the identified compounds. NM: nonmutagen; NC: noncarcinogen; C: carcinogen; NI: nonirritant; NS: nonsensitive; S: sensitive. [file 1297484.f1.pdf]

Supplementary files

Fig. S1: Diagrammatic representation of Structure-guided approach to design potential inhibitors of LHBs protein.

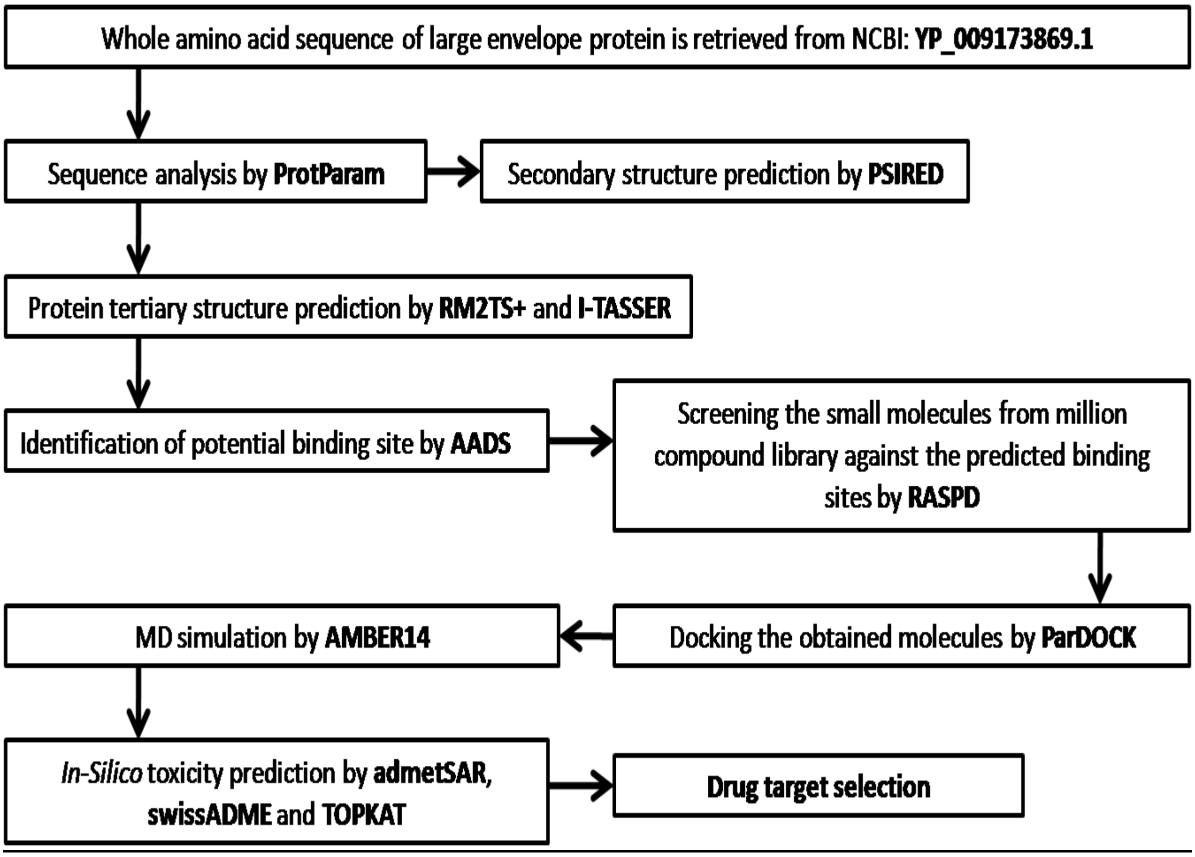

Fig. S2: Distribution of α-helix (pink), β-sheet (yellow) and coil (black) in the LHBs protein

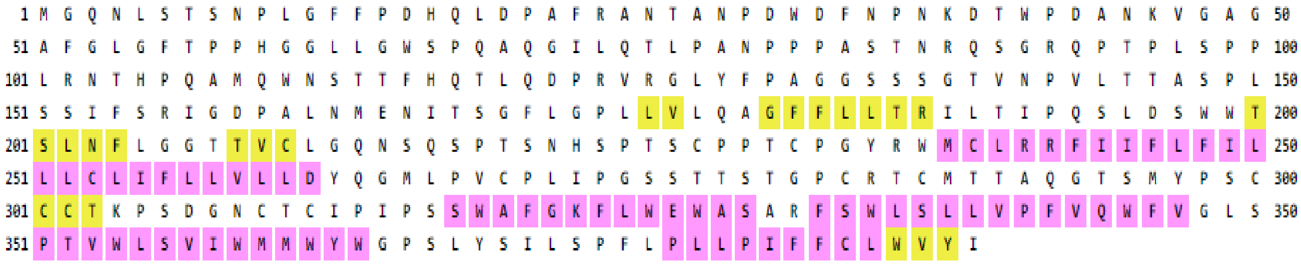

Fig S3: The structures which were obtained from RM2TS+ and I-TASSER were further analyzed using various tools such as ProtSAV, Protein Structure Analysis and Validation, but for getting better structure the MD was run for 20 ns.

- **LHBs structure before MD**

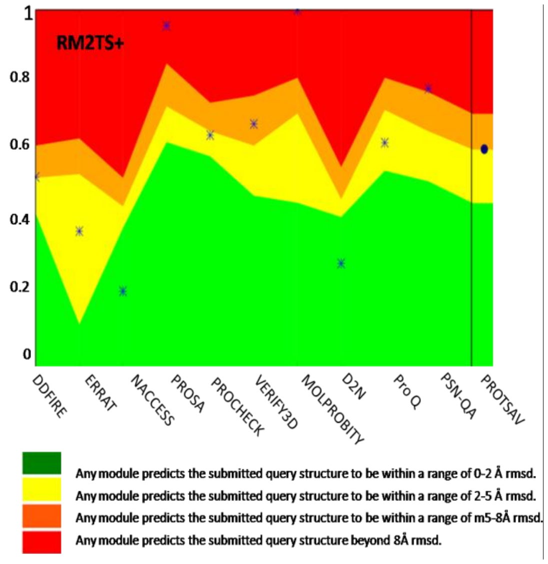

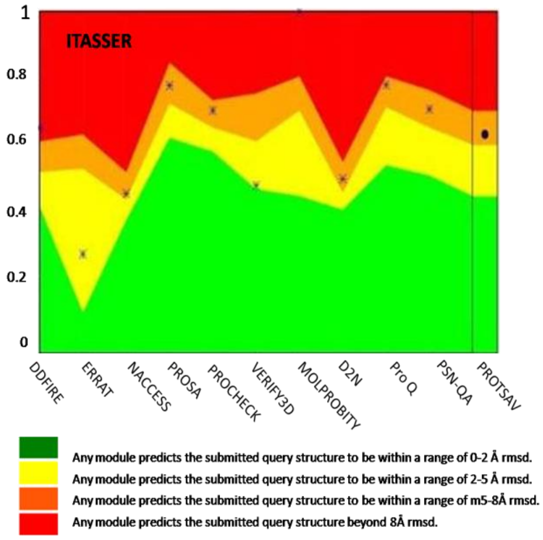

- **LHBs structure after MD**

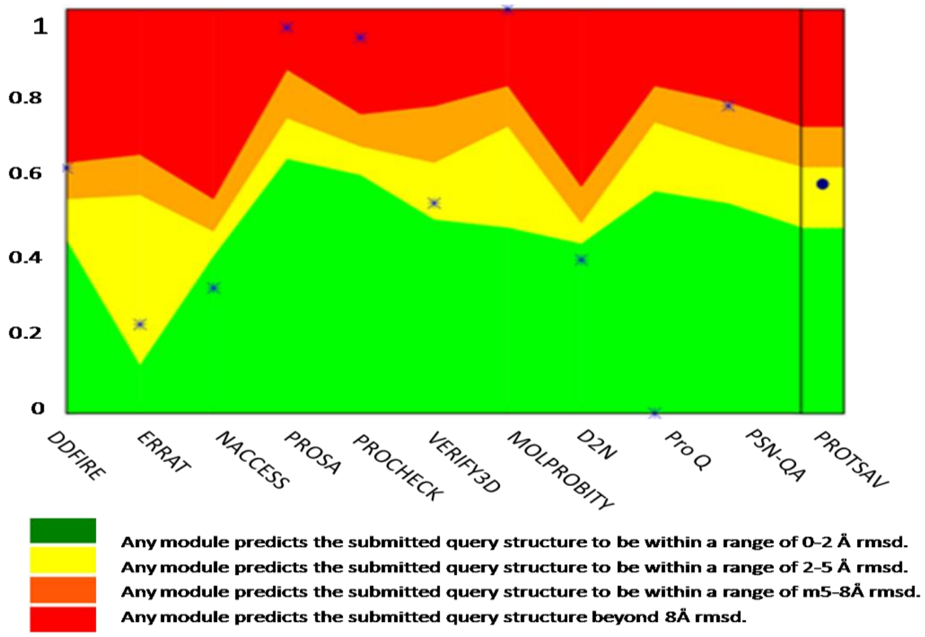

Table S1: Physiological properties of LHBs protein**.**

Protein	Molecular wt.(Da)	Theoretical pI	Estimated half-life	Instability index	Aliphatic index	No. of amino acid	No. of atoms	GRAVY
LHBs	42766.45	8.40	30hrs	46.08	82.24	389	5967	0.146

Table S2: 2D structure of identified ligands from ZINC database

No	ZINC ID	ParDock ID	2D Structure of Ligand
1	11882026	33625775	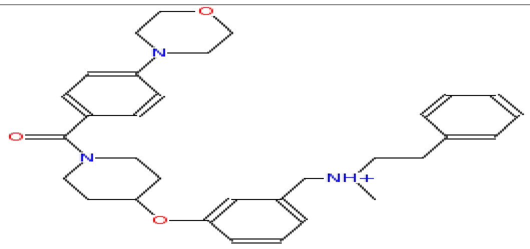

2	653293	21425236	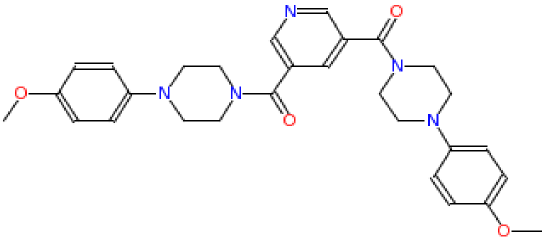
3	19741044	72860345	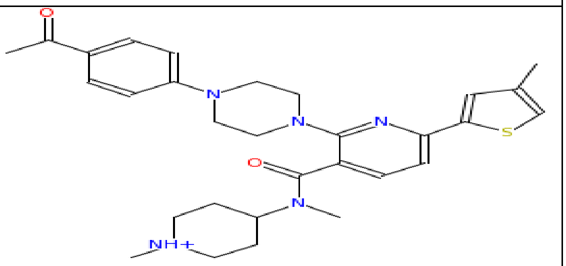
4	15000762	78876711	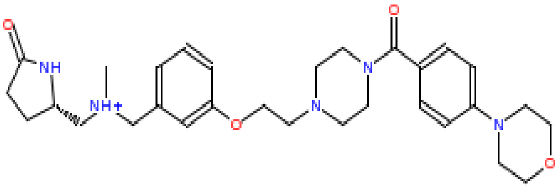
5	11784805	34135187	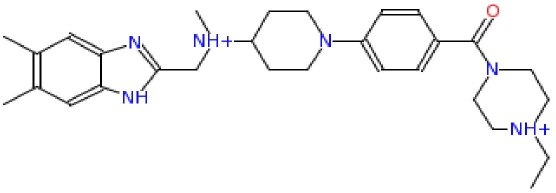
6	12243260	32993271	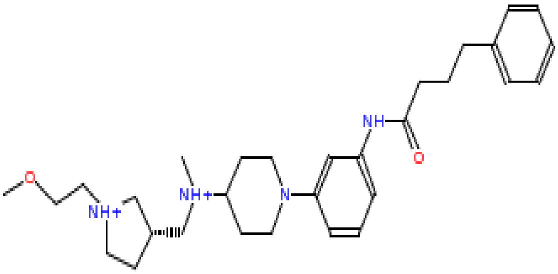

Table S3: Comparative analysis of hydrogen bonds in LHBs and four ligands.

NO	ZINC ID	Receptor atom (LHBs)	Ligand atom	No. of H bonds	Bonding Length (Ǎ)
1	11882026	Ala (51) [O]<br>Trp (111) [O]<br>Thr (104) [O]	[N]<br>[N]<br>[O]	3	3.33<br>3.08<br>3.32
2	653293	Pro (106) [O]<br>Ala (108) [N]	[N]<br>[N]	2	2.9<br>3.2
3	19741044	Ser (90) [N]	[O]	1	3.20
4	15000762	Ser (280) [N]	[O]	1	2.89

Table S4: Computationally predicted ADME properties of the identified compounds 1-5 by admetSAR and SwissADME. NS: (Non- Substrate); S: (Substrate); NI: (Non-Inhibitor); NT: (Non-Toxicity); NC: (Non-Carcinogen); NR: (Non-required)

**Descriptor**	ZINC11882026	ZINC00653293	ZINC19741044	ZINC15000762
Absorption				
Blood-Brain Barrier	BBB+	BBB+	BBB+	BBB+
Human Intestinal Absorption	HIA+	HIA+	HIA+	HIA+
GI - absorption	High	High	High	High
Caco-2 permeability	Caco2+	Caco2+	Caco2+	Caco2+
Metabolism				
CYP4502C9 Substrate	NS	NS	NS	NS
CYP4502D6 Substrate	S	NS	NS	NS
CYP4503A4 Substrate	S	S	S	S
CYP4501A2 Inhibitor	NI	NI	NI	NI

CYP4502C9 Inhibitor	NI	I	I	I
CYP4502D6 Inhibitor	NI	I	NI	NI
CYP4502C19 Inhibitor	I	I	I	NI
CYP4503A4 Inhibitor	NI	I	NI	NI
Toxicity				
AMES Toxicity	NT	NT	NT	NT
Carcinogens	NC	NC	NC	NC
Carcinogenicity (Three-class)	NR	NR	NR	NR
**Number of drug likeliness rules followed***				
Lipinski	Yes	Yes	Yes	Yes
Ghose	No	No	No	Yes
Veber	Yes	Yes	Yes	Yes
Egan	Yes	Yes	Yes	Yes
Muegge	No	Yes	Yes	Yes

Table S5: TOPKAT values of the identified compounds. NM: (Non-Mutagen); NC: (Non-Carcinogen); C: (Carcinogen); NI: (Non- irritant); NS: (Non-Sensitive); S: (Sensitive)

**Descriptor**	ZINC11882026	ZINC00653293	ZINC19741044	ZINC15000762
Ames Mutagenicity (v3.1)	NM (0.853)	NM (0.000)	NM (0.000)	NM (0.717)
Weight of Evidence Carcinogenicity Call (v5.1)- (WOE)	NC (0.000)	NC (0.394)	C (1.000)	NC (0.003)
Rat Oral LD50 (v3.1)	2.432	3.946	4.559	2.895
Skin Irritation (v6.1)	NI (0.000)	NI (0.000)	NI (0.000)	NI (0.000)
Skin Sensitization NEG v SENS (v6.1)	NS (0.493)	NS (0.000)	NS (0.088)	NS (0.000)
Aerobic Biodegradability (v6.1)	No	Yes	No	Yes
